# Correlation between Compartmental Tenofovir Concentrations and an *Ex Vivo* Rectal Biopsy Model of Tissue Infectibility in the RMP-02/MTN-006 Phase 1 Study

**DOI:** 10.1371/journal.pone.0111507

**Published:** 2014-10-28

**Authors:** Nicola Richardson-Harman, Craig W. Hendrix, Namandjé N. Bumpus, Christine Mauck, Ross D. Cranston, Kuo Yang, Julie Elliott, Karen Tanner, Ian McGowan, Angela Kashuba, Peter A. Anton

**Affiliations:** 1 Alpha StatConsult, LLC, Damascus, MD, United States of America; 2 Departments of Medicine and Pharmacology, Johns Hopkins University School of Medicine, Baltimore, MD, United States of America; 3 CONRAD, Arlington, VA, United States of America; 4 Department of Medicine, University of Pittsburgh, Pittsburgh, PA, United States of America; 5 Magee-Womens Research Institute, University of Pittsburgh Medical School, Pittsburgh, PA, United States of America; 6 Center for HIV Prevention Research, UCLA AIDS Institute, Department of Medicine, David Geffen School of Medicine at UCLA, Los Angeles, CA, United States of America; 7 Eshelman School of Pharmacy, University of North Carolina at Chapel Hill, Chapel Hill, NC, United States of America; Imperial College London, United Kingdom

## Abstract

**Objectives:**

This study was designed to assess the dose-response relationship between tissue, blood, vaginal and rectal compartment concentrations of tenofovir (TFV) and tenofovir diphosphate (TFVdp) and *ex vivo* rectal HIV suppression following oral tenofovir disoproxil fumarate (TDF) and rectal administration of TFV 1% vaginally-formulated gel.

**Design:**

Phase 1, randomized, two-site (US), double-blind, placebo-controlled study of sexually-abstinent males and females.

**Methods:**

Eighteen participants received a single 300 mg exposure of oral TDF and were then randomized 2∶1 to receive a single then seven-daily rectal exposures of TFV 1% gel (40 mg TFV per 4 ml gel application) or hydroxyethyl-cellulose (HEC) placebo gel. Blood and rectal biopsies were collected for pharmacokinetic TDF and TFVdp analyses and *ex vivo* HIV-1 challenge.

**Results:**

There was a significant fit for the TFVdp dose-response model for rectal tissue (*p* = 0.0004), CD4^+^_MMC_ (*p*<0.0001), CD4^−^_MMC_ (*p*<0.0001), and Total_MMC_ (*p*<0.0001) compartments with *r^2^* ranging 0.36–0.64. Higher concentrations of TFVdp corresponded with lower p24, consistent with drug-mediated virus suppression. The single oral treatment failed to provide adequate compartment drug exposure to reach the EC_50_ of rectal tissue TFVdp predicted to be necessary to suppress HIV in rectal tissue. The EC_50_ for CD4^+^_MMC_ was within the single topical treatment range, providing evidence that a 1% topical, vaginally-formulated TFV gel provided *in-vivo* doses predicted to provide for 50% efficacy in the *ex vivo* assay. The 7-daily topical TFV gel treatment provided TFVdp concentrations that reached EC_90_ biopsy efficacy for CD4^−^_MMC_, CD4^+^_MMC_ and Total_MMC_ compartments.

**Conclusion:**

The TFVdp MMC compartment (CD4+, CD4− and Total) provided the best surrogate for biopsy infectibility and the 7-daily topical TFV gel treatment provided the strongest PK profile for HIV suppression.

ClinicalTrials.gov NCT00984971.

## Introduction

*Ex vivo* infection of rectal and cervical biopsies has been used as a potential biomarker of microbicide efficacy in humans. *Ex vivo* tissue biopsies are infected with HIV, following either *ex vivo* [1]–[5] or, more recently, *in vivo* [6], [7] exposure to a topical microbicide product. When HIV suppression in the biopsy infectibility assay correlates with drug concentration in tissue it is possible to derive tissue drug concentrations predicting 50–95% suppression of *ex vivo* infections [8]. In the currently reported RMP-02/MTN-006 Phase 1 rectal microbicide trial of topical and oral TFV, drug concentrations were quantified in multiple compartments (blood, rectal tissue, rectal/vaginal fluid compartment concentrations; ‘CC’) and correlated with HIV suppression in the *ex vivo* infectibility rectal tissue assay to provide a measure of drug efficacy.

In the first rectal microbicide trial to correlate *ex vivo* tissue infectibility with tissue drug concentration following *in vivo* application of a product (0.1 or 0.25% UC781 gel [8]), logistic regression was used to calculate the EC_50,90,95_ tissue concentrations predicted to result in 50, 90 or 95% biopsy non-infectibility. In this analysis model, it was shown that defining a tissue sample as either infected or non-infected enabled a predictive dose-response relationship to be identified. Tissue infection was indicated when HIV replication in the *ex vivo* assay was greater than 500 pg/mL cumulative p24 on Day 14. Although there is currently no consensus on the optimal method for p24 quantification in the *ex vivo* challenge assay, cumulative p24 on Day 14 has been found to be a relatively reliable and precise method for quantifying rectal *ex vivo* HIV replication [6]–[8].

The RMP-02/MTN-006 clinical trial evaluated the safety (primary outcome), acceptability, pharmacokinetic (PK; secondary outcome), pharmacodynamic profile (PD; exploratory outcome) and a limited PK:PD analysis of tissue TFVdp and biopsy p24 ([6]) of TVF 1% gel (single and 7-daily exposures) and oral tenofovir disoproxil fumarate (TDF; 300 mg; single exposure). Single oral and topical exposures provided the opportunity to assess within-subject comparisons of safety, PK and PD following single product exposure. Safety, PK and PD assessment was also conducted after 7 doses of topical TFV gel, a period approaching the time needed to reach steady state levels of TFV. The objective of this analysis was to report on the RMP-02/MTN-006 [6] multi-compartment correlations between PK (i.e. TFV) and tenofovir diphosphate (TFVdp) compartment drug concentrations and PD activity (i.e. biopsy infectibility reflected by the degree of *ex vivo* p24 suppression in tissue biopsies).

## Methods

### Study participants

**Figure 1:**
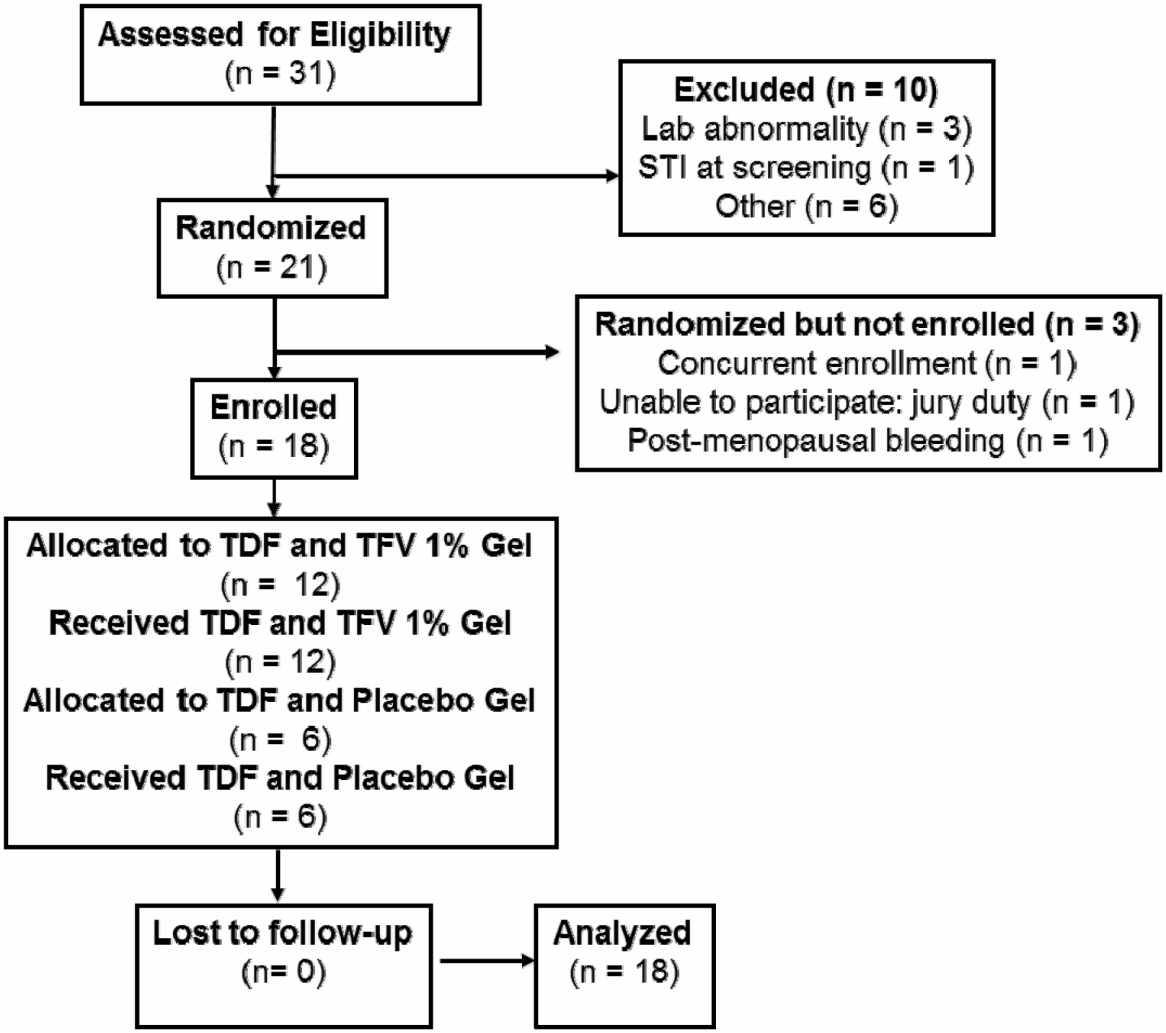
CONSORT flowchart.

**Figure 2:**
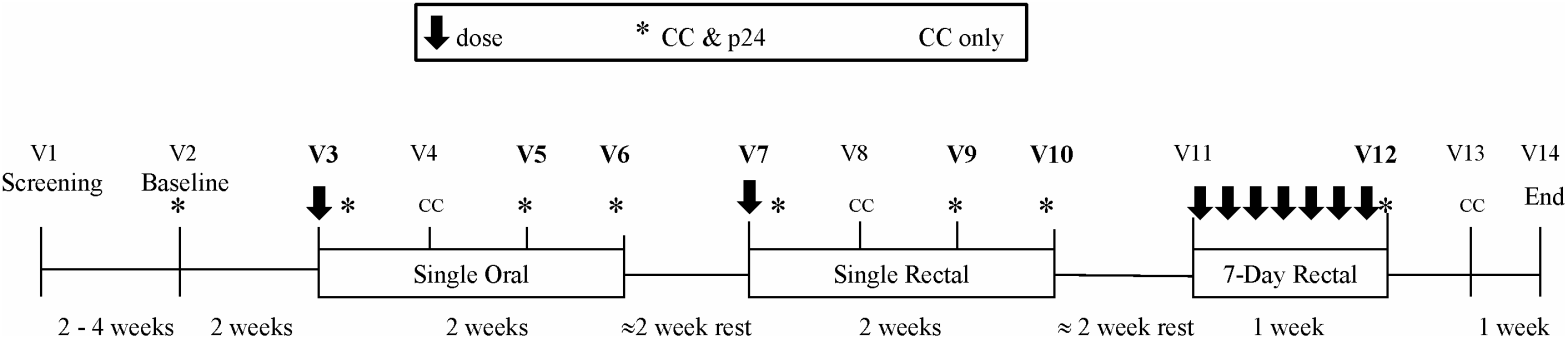
Study flow diagram. Paired measures from compartment concentrations (CC) and both CC and biopsy samples (*) taken at the bolded visits: V3 (Visit 3: ∼30 mins post single oral dose), V5 (1–6 days post V3 dose), V6 (7–9 days post V3 dose), V7 (∼30 mins post single topical dose), V9 (1–3 days post V7 dose), V10 (7–12 days post V7 does) and V12 (∼30 mins post 7^th^ daily dose) used in the dose-response analysis.

Study participants (N = 18; Figure 1) were healthy HIV-1 seronegative males and females with a history of consensual receptive anal intercourse (RAI), willing to abstain from vaginal and anal sex during active protocol phases (V2–V14, Figure 2). Female participants were required to use an acceptable form of contraception.

### Ethics statement

The trial was IRB-approved at each site (UCLA, Los Angeles, CA; University of Pittsburgh, Pittsburgh, PA); all participants provided written informed consent. RMP-02/MTN-006 is registered at ClinicalTrials.gov (#NCT00984971) and is in compliance with the CONSORT 2010 trial reporting recommendations (www.consortstatement.org). The protocol for this trial and supporting CONSORT checklist are available as supporting information; see Checklist S1 and Protocol S1.

### Study design

The design of RMP-02/MTN-006 Phase 1 trial has been described [6] and is briefly outlined here. This was a double-blind, randomized, placebo-controlled comparison of oral TDF (300 mg), rectally-applied TFV 1% gel (each dose of gel contained 40 mg of TFV), and the HEC placebo gel. Randomization was carried out by the study pharmacist and was a two-part process. Following enrollment, participants were randomized (2∶1) to receive TFV 1% gel (N = 12) or HEC placebo gel (N = 6). A separate randomization was used to assign subjects, for safety reasons, into one of two post-exposure biopsy sampling arms (A or B) after each single exposure stage. Group A subjects were biopsied on Days 1–3 and 7–9 and Group B subjects were biopsied on Days 4–6 and 10–12. Each two week period of biopsy sampling was followed by a two week washout period between stages (‘rest’; Figure 2). At visit 3 (V3 N = 18; Figure 2), all participants received a single oral dose of TDF administered by a clinical team; this was followed 4-weeks later (V7) by a single dose of rectally applied product administered by the clinical team. Four weeks later, participants received seven sequential daily doses of their assigned products; six of which were self-administered each morning with the 7^th^ rectal dose administered in the clinic by the clinical team (V12; Figure 2). Rectal biopsy infectibility assays were repeatedly performed over 2 weeks following each treatment phase, with concurrent CC (i.e. compartment concentrations) measurements of: (i) TFV from rectal and vaginal fluids, blood and rectal tissue and; (ii) TFVdp from total peripheral blood mononuclear cells (Total_PBMC_), CD4+ lymphocytes from PBMC (CD4^+^_PBMC_), CD4− lymphocytes from PBMC (CD4^−^_PBMC_)] and tissue [whole tissue biopsy, total isolated mucosal mononuclear cells (Total_MMC_), CD4+ lymphocytes from MMC (CD4^+^_MMC_), CD4− lymphocytes from MMC (CD4^−^_MMC_)]. CC and explant tissue samples were taken at time points from 30 minutes through 14 days following each single exposure (oral and topical) and at 30 minutes following the 7-day exposure. Biopsy infectibility and compartmental PK measures were obtained at 7 timepoints following each product exposure (from 30 minutes to 12 days; e.g., V3, 5, 6, 7, 9, 10 and 12, ‘*’; Figure 2). These paired (blood/biopsy) timepoints were included in this dose-response analysis. CC and biopsy samples taken from all participants at baseline (V2; no drug exposure) as well as from those participants randomized to the placebo arm (N = 6; V7, 9, 10 and 12) did not follow drug exposure and so were not included in the dose-response analysis. Sample size (n = 18) was based on similar phase 1 studies of topical microbicides [7]. Enrollment began November 2009 and was completed July 2010. Baseline demographic and clinical characteristics for each group are reported in the original trial paper [6].

### Study products

300 mg tablets of TDF were supplied by Gilead Sciences Inc. (Foster City, CA). TFV 1% gel providing 40 mg TFV per 4 mL application, and HEC gel were supplied by CONRAD (Arlington, VA). The vaginal formulation of TFV 1% gel used was pH adjusted to 4–5 with an osmolarity of 3111 mOsmol/kg and the HEC placebo was isotonic with a pH of 4.4, osmolarity of 304 mOsmol/kg [6] and a viscosity similar to other microbicide gel candidates [9]. TFV and HEC gels were pre-filled into single-use, opaque applicators (HTI Plastics; Lincoln, NE) containing approximately 4 mL of gel.

### PK analyses

Plasma TFV and tissue TFVdp concentrations were determined by previously described LC-MS/MS methods validated for all matrices at The Johns Hopkins Clinical Pharmacology Analytical Laboratory and met FDA bioanalytical validation criteria [10]. TFV concentrations were determined in both peripheral blood mononuclear cells (PBMC) and mucosal mononuclear cells (MMC) for rectal and vaginal fluids (ng/sponge; Ultracell Aspen, Caledonia MI) in addition to plasma (ng/mL) and tissue (ng/mg) concentrations. TFVdp concentrations were also determined for Total_PBMC_, CD4^+^_PBMC_, CD4^−^_PBMC_, Total_MMC_, CD4^+^_MMC_, CD4^−^_MMC_ and Tissue. CD4^+^ and CD4^−^ subsets from both PBMC and MMC were acquired by MACS Miltenyi Biotec QuadroMACS separation unit (Miltenyi Biotec Inc., Auburn, CA). The measured value from each PK assay was used unless the PK value was determined to be between the lower limit of quantification (LLOQ) and the lower limit of detection (LLOD). In these cases, a number equal to half that assay’s LLOQ was imputed for that PK value.

### PD analyses

At baseline (V2) and timepoints following product exposure (V3, 5, 6, 7, 9, 10 and 12; Figure 2), endoscopic biopsies were collected in 50 mL RPMI (with 1.125 µg/mL of Fungizone and 50 mg/mL of Zosyn) and transported to the laboratory for *ex vivo* infection within ∼1–2 hours using a common viral stock of HIV-1_BaL_ (10^4^ TCID_50_), as previously described [1], [11], [12]. Supernatants for p24 quantification were collected every three days during each 14-day infectibility assay (Days 1, 4, 7, 11 & 14). Results were averaged across quadruplicate assays and reported as cumulative p24 (p24 HIV antigen ELISA; NCI, Bethesda, MD) where the assay’s LLOQ was 10 p24 pg/mL. Non-detectable cumulative p24 measures at Day 14 were converted to 1/2 the LLOQ prior to log transformation. Cumulative p24 was used here to provide both a continuous (i.e. pg/mL) and binary (<500 pg/mL cumulative p24 = ‘non-infected’; ≥500 p24 pg/mL = ‘infected’) measure of virus growth [8], [13].

### Statistical analysis

TFV measures from four compartments (rectal fluid, vaginal fluid, plasma, and rectal tissue) and TFVdp measures from seven compartments (Total_PBMC_, CD4^+^_PBMC_, CD4^−^_PBMC_, Total_MMC,_ CD4^+^_MMC_, CD4^−^_MMC_, and rectal tissue) were log_10_ transformed and paired with the corresponding log transformed explant infectibility result (i.e. log_10_ cumulative p24 at Day 14) for each subject and sampling time. The number of CC and p24 paired measurements (N = 18) are reported in Table 1. Paired CC and p24 endpoints were entered into a three parameter, log-log, Hill slope, non-linear model (1) where the fit of the model was tested by nonlinear least-squares ANOVA and the proportion of variance that each model explained (*r^2^*) was calculated [i.e. (1−) the sum of the squared distances from each fitted curved divided by the squared distances from a horizontal line].

**Table 1:** Number of detectable, concurrent, paired CC and p24 measurements following each treatment.

Compartment	Single Oral*	Single Topical**	7-Daily Topical***	Total
TFV Rectal Fluid	38	27	12	77
TFV Vaginal Fluid	6	1	2	9
TFV Plasma	45	16	12	73
TFV Rectal Tissue	14	10	10	34
TFVdp Rectal Tissue	10	17	12	39
TFVdp CD4^−^_MMC_	17	17	11	45
TFVdp CD4^+^_MMC_	10	13	9	32
TFVdp Total_MMC_	4	14	9	27
TFVdp CD4^−^_PBMC_	9	1	0	10

*Post Single Oral (V3, 5 & 6). Maximum of 18 (N; number of subjects)×3 (V; visits) = 54 paired CC:p24 measurements, 12 for vaginal fluid [4 (f; female subjects)×3 (V)].

**Post Single Topical (V7, 9 &10). Maximum of 12 (N)×3 (V) = 36 paired CC:p24 measurements,6 for vaginal fluid [2(f)×3 (V)].

***Post 7-Daily Topical (V12). Maximum of 12 (N)×1 (V) = 12 paired CC:p24 measurements, 2 for vaginal fluid [2(f)×1 (V)].



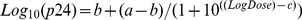
(1)The fit of each three-parameter non-linear model was compared to an alternative four parameter model using the information criterion of Akaike (AIC), where a lower AIC indicates improved model fit [14].

The ability of a microbicide treatment to suppress p24 in the *ex-vivo* assay is an indication of treatment efficacy, where lower concentrations of p24 indicate virus suppression. Based on earlier published methodology [8], biopsy assays resulting in a cumulative p24 below 500 pg/mL were categorized as ‘suppressed’ and biopsy assays with a cumulative p24 at or above 500 pg/mL were ‘not-suppressed.’ This binary categorization of *ex-vivo* endpoints (i.e. ‘suppressed’ or ‘not suppressed’) was then used in a logistic regression model of the relationship between probability of *ex vivo* HIV suppression and CCs. CCs necessary to suppress 50, 90 and 95% of biopsy HIV (i.e. EC_50_, EC_90_, and EC_95_) were calculated by interpolation of the logistic curve at 0.50, 0.90 and 0.95 probability. A predicted EC_50_, EC_90_, or EC_95_ CC that fell within the PK range (min-max) found following each treatment [15] was evidence that the treatment provided a CC with the potential to meet EC_50,90,95_ levels of efficacy.

All statistical analyses were performed using SAS/STAT software Version 9.3 of the SAS system for Windows (SAS Institute Inc., Cary, NC) and an alpha = 0.05.

## Results

Paired CC and p24 data meeting the following criteria were included in the analysis: (i) collected post active drug, (ii) at timepoints with concurrent *ex vivo* infectibility assays and, (iii) with measurable CC and p24 concentrations. For example, of the 102 possible rectal fluid TFV and p24 paired measurements following the single oral (N = 18 receiving active drug), single topical (N = 12) and 7-daily topical exposure stages, there were, respectively, 38, 27 and 12 HIV *ex vivo* p24 measures that were paired with detectable rectal fluid TFV CCs (‘TFV Rectal Fluid; Table 1; see File S1 for non-detectable and missing data frequencies). There were no detectable CC measures of TFVdp from either Total_PBMC_ or CD4^+^_PBMC_ at the time-points when biopsies were acquired (‘*’, Figure 2); no further analyses were performed on these compartments.

### Compartment drug-HIV suppression correlations

The non-linear model fit was improved (smaller AIC) for the three parameter, compared to the four parameter, regression model that was used to correlate TFV and TFVdp CCs with tissue infectibility (cumulative p24; Table 2).

**Table 2:** TFV and TFVdp dose, p24 suppression response non-linear models.

Compartment	n	*p*	*r^2^*	AIC* (3-param§)	AIC (4-param§§)
TFV Rectal Fluid (ng/sponge)	77	0.0012	0.17	149.29	151.29
TFV Vaginal Fluid (ng/sponge)	9	ns	0.26	27.83	29.83
TFV Plasma (ng/mL)	73	ns	0.01	155.84	157.84
TFV Rectal Tissue (ng/mg)	34	ns	0.13	81.41	83.41
TFVdp Rectal Tissue (fmol/mg)	39	0.0004	0.36	81.96	83.96
TFVdp CD4^−^_MMC_ (fmol/10^6^ cells)	45	<0.0001	0.64	69.42	71.42
TFVdp CD4^+^_MMC_ (fmol/10^6^ cells)	32	<0.0001	0.53	63.57	65.57
TFVdp Total_MMC_ (fmol/10^6^ cells)	27	<0.0001	0.57	55.55	57.55
TFVdp CD4^−^_PBMC_ (fmol/10^6^ cells)	10	ns	0.46	14.84	16.84

*p* = probability of non-linear model fit.

ns = non-significant at alpha 0.05.

n = number of CC:p24 paired measurements.

*r^2^* = (1-) the sum of the squared distances from each fitted curved divided by the squared distances from a horizontal line.

*AIC = Akaike information criterion value. The 3-parameter non-linear model provided lower AIC values indicating a better fit than an alternative 4-parameter model.

§ 3-parameter model: 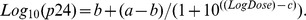

§§ 4-parameter model: 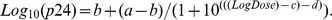

**Figure 3:**
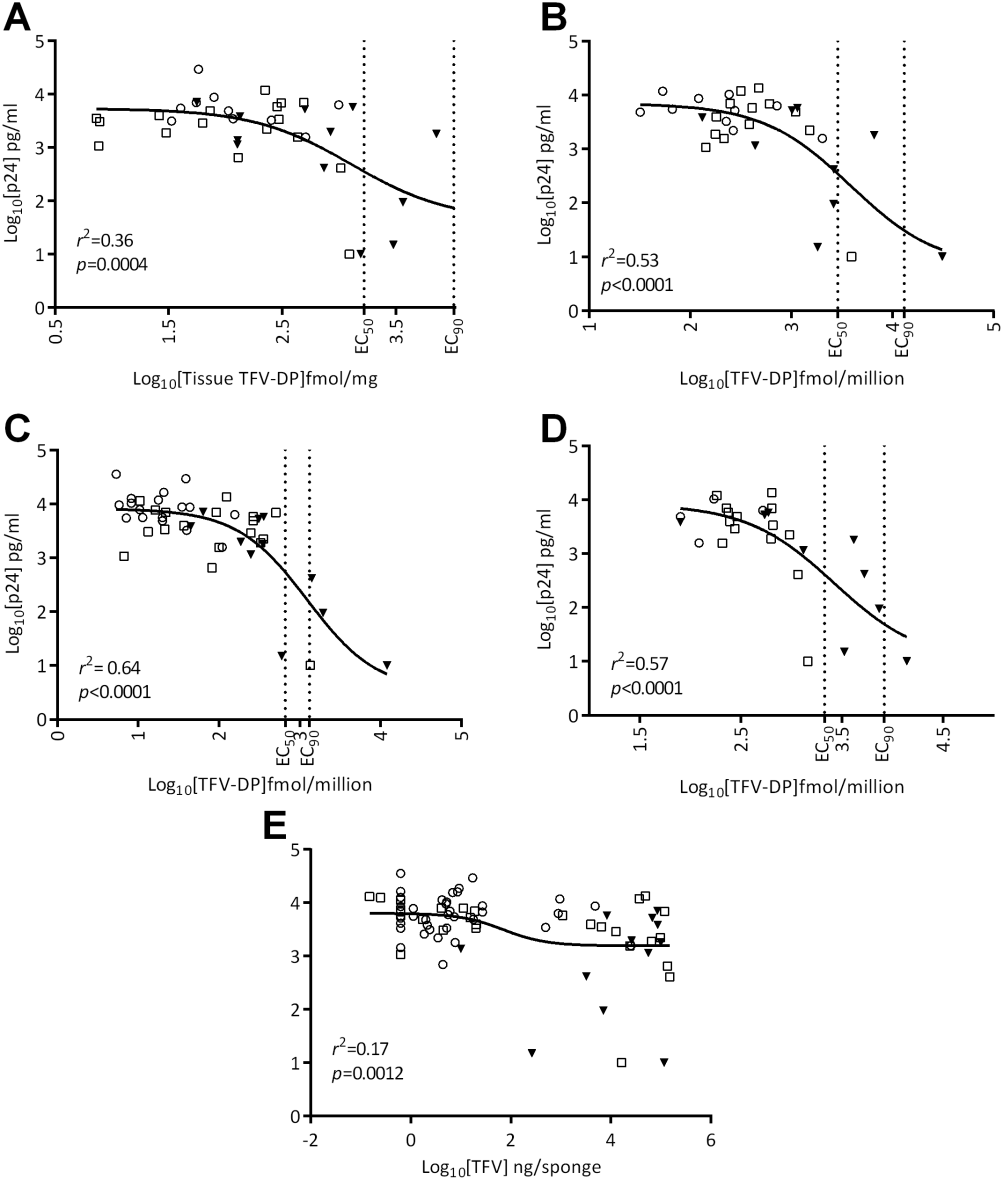
Tenofovir (TFV) and Tenofovir Diphosphate (TFVdp) concentration and biopsy cumulative p24 dose-response relationships. Results are shown for those CC:p24 paired measurements with detectable concentrations of drug following single oral TDF (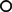), single topical TFV gel (□) and 7-day topical TFV gel (▾) microbicide treatments for the dose-response relationship between CC drug concentrations and *ex vivo* p24 levels. Figure panels A-D show TFVdp in (A) rectal tissue, (B) TFVdp CD4^+^_MMC,_ (C) TFVdp CD4^−^_MMC_, (D) TFVdp Total_MMC_ and (E) Tenofovir (TFV) rectal fluid concentration. Parameters from the log-log non-linear model with a Hill slope factor of –1.0, where *r* ^2^ and probability levels for the fit of the non-linear least-squares analysis of variance are embedded. Vertical lines indicate the EC_50_ and EC_90_ calculated by the logistic regression analysis as the compartmental drug concentrations predicted to provide 50 and 90% efficacy in the explant infectivity assay.

TFVdp*:* There was a significant fit by non-linear least squares analysis of variance, for the TFVdp dose-response model for rectal tissue (*p* = 0.0004; Figure 3a.), CD4^+^_MMC_ (*p*<0.0001; Figure 3b.), CD4^−^_MMC_ (*p*<0.0001; Figure 3c.), Total_MMC_ (*p*<0.0001; Figure 3d.) compartments with *r^2^* ranging 0.36–0.64. Higher concentrations of TFVdp corresponded with lower p24, consistent with drug mediated virus suppression. Non-linear curves provided a clear upper asymptote, where lower ranges of drug were ineffective in suppressing virus growth (Figures 3a–d). There was little or no evidence of a lower asymptote, where suppression of HIV reached the lower limit of p24 quantification and additional drug was not increasingly efficacious (Figure 3a–d). No significant CC:p24 relationships were identified in the blood-derived samples of CD4^−^_PBMC_ (Table 2) at the PK timepoints that were concurrent with an endoscopic biopsy procedure.

TFV: There was a significant fit for the non-linear model of rectal fluid TFV and p24 biopsy suppression (Figure 3e; *p* = 0.0012); higher concentrations of TFV correlated with lower cumulative p24. Although statistically significant, this rectal fluid TFV CC:p24 suppression model had a relatively low *r^2^* value (0.17). This is likely reflective of many high CC values [∼4–6 log_10_(TFV) ng/sponge] not associated with suppression of biopsy p24 [∼3–4 log_10_(p24) pg/mL; Figure 3e]. No significant CC:p24 relationships were identified for TFV in vaginal fluid, plasma or rectal tissue, the latter in contrast to the significant relationship found for TFV in rectal fluid (Table 2).

### TFVdp and TFV EC_50,90,95_

Logistic regression analyses were performed for those TFV and TFVdp compartments showing significant dose-response relationships: TFVdp concentrations in rectal tissue, CD4^+^_MMC_, CD4^−^_MMC_, Total_MMC_ and TFV concentrations in rectal fluid (Figure 3a–e). The concentrations of drug (TFV or TFVdp) in each compartment predicted to suppress biopsy HIV below a cumulative p24 of 500 pg/mL were determined using a logistic regression model. The fit of the logistic regression model was measured by the area under the receiver operator characteristic curve (‘AUC’) where an AUC = 1.0 indicates perfect prediction of biopsy suppression [16]. The fit of the logistic models ranged from 0.83–1.00 AUC. Interpolation at the 50, 90 and 95% HIV suppression probability levels of the logistic curve provided EC_50,90,95_ point estimates for TFV and TFVdp CCs (Table 3). Bootstrap samples (x1000, data not shown) were run and results were highly consistent at the EC_50_ endpoint (within 4% of the Table 3 EC_50_ values) but less consistent at the EC_90_ and EC_95_ efficacy levels presumably due to few observations at those modeled concentrations.

**Table 3:** Compartment TFV and TFVdp efficacy concentrations (EC_50,90,95_) predicted by logistic regression to suppress HIV infection following single oral TDF, single topical TFV 1% gel and 7-day topical TFV 1% gel *in vivo* product use.

Compartment (measured unit)	Predicted Efficacy§		
	EC_50_	EC_90_	EC_95_
TFV Rectal Fluid (ng/sponge)	4.9×10^6^	3.6×10^10^	3.4×10^11^
TFVdp CD4^−^_MMC_ (fmol/10^6^ cells)	661	1318	1549
TFVdp CD4^+^_MMC_ (fmol/10^6^ cells)	2884	13183	19498
TFVdp Total_MMC_ (fmol/10^6^ cells)	2138	8318	12023
TFVdp Rectal Tissue (fmol/mg)	1660	10233	16596

§ Predicted compartment dose concentrations to suppress 50, 90 and 95% of HIV infection interpolated from the logistic regression probability curve where infection was defined as cumulative p24 pg/mL ≥500.

Actual drug concentrations in the delivered product were 300 mg tenofovir disoproxil fumarate in the oral pill (equivalent to 245 mg of tenofovir disoproxil) and 40 mg/4 mL tenofovir in each topical gel application.

The likelihood that a treatment (i.e. single oral TDF, single topical TFV gel or 7-daily topical TFV gel) would provide a CC necessary for *ex vivo* HIV suppression (i.e. EC_50,90,95_) was evaluated by determining whether the EC_50,90,95_ point estimates were within the range found following each treatment ([15]; Table 2). An EC_50,90_ and/or EC_95_ estimate that fell within the likely range found in subjects following a treatment provided evidence that the treatment had the potential to suppress biopsy HIV growth at the 50, 90, or 95% probability level. Conversely, if the range in actual CC following a treatment was less than the predicted EC_50,90,95_ levels then the treatment failed to provide CC at levels predicted to be necessary for suppression of HIV growth in the biopsy assay.

The single oral treatment failed to provide adequate CC to reach the EC_50_ levels of rectal tissue TFVdp predicted to be necessary to suppress HIV in the rectal biopsy [ie. TFVdp (fmol/mg) = 1660; Table 3] at 24 hours post treatment (i.e. TFVdp C_24 hrs_ (fmol/mg) = BLQ-991; min-max; [15]). The EC_50_ level for CD4^+^_MMC_ (2884 fmol/10^6^ cells; Table 3) was within the single topical treatment range at C_30 min_ (BLQ-3950 fmol/10^6^ cells; [15]), providing evidence that a TFV 1% gel could deliver a CD4^+^_MMC_ concentration necessary for 50% efficacy in the *ex vivo* assay. The 7-daily topical TFV gel treatment provided TFVdp concentrations (min-max) that reached EC_90_ biopsy efficacy for CD4^−^_MMC_ (C_30 min_ = BLQ-12000 fmol/10^6^ cells; Table 2 [15]; EC_90_ = 1318 fmol/10^6^ cells Table 3), CD4^+^_MMC_ (C_30 min_ = BLQ-31200 fmol/10^6^ cells [15]; EC_90_ = 13183 fmol/10^6^ cells Table 3) and Total_MMC_ (C_30 min_ = BLQ-13900 fmol/10^6^ cells [15]; EC_90_ = 8318 fmol/10^6^ cells Table 3) compartments. The EC_50,90,95_ serve as point estimates of efficacy where the range in *in vivo* drug concentrations found following use of an efficacious product would, ideally, be higher than the EC_50,90,95′_s found in this *ex vivo* model. Up to a 4-log spread in compartment drug concentration was found following the single oral, single rectal and 7 day rectal dosing, where only a small proportion of subject timepoints resulted in concentrations exceeding the predicted EC_90_ doses. For example, only 3/12 of detectable CD4^−^_MMC_ concentrations of TFVdp were above the EC_90_ of 1318 fmol/10^6^ cells (Figure 3c), providing evidence suggestive of at least partial efficacy in this model.

## Discussion

This Phase 1 trial confirmed that the *ex vivo* biopsy challenge model can provide a pharmacodynamic endpoint (p24 suppression) correlated with *in vivo* drug concentrations and varied by treatment regimen. The pharmacodynamic endpoint of p24 suppression following HIV-1_BaL_ infection of freshly-acquired human tissue biopsies has yet to be validated as a bio-indicator of HIV prevention in a large scale human efficacy trial but does present the closest surrogate currently available. This paper demonstrated that tissue HIV infectibility (cumulative p24) was inversely correlated with *in vivo* concentrations of both TFV and TFVdp. Statistically significant, non-linear dose-response relationships with reduced tissue infectibility were found for one TFV compartment and four TFVdp compartments; the dose-response relationships were highly significant for TFVdp in whole rectal tissue, CD4^+^_MMC_, CD4^−^_MMC_ and Total_MMC_ compartments. The finding that TFVdp, the active metabolite of TFV, was more predictive of *ex vivo* virus inhibition than TFV demonstrates the utility of measuring TFVdp in cellular spaces. Isolation of cells for the measurement of TFVdp may not be necessary as TFVdp in whole rectal tissue was found to be as predictive of a pharmacologic response as TFVdp in isolated cell populations. These findings support the measurement of TFVdp from whole tissue homogenates in clinical trials, as isolation of cells from tissue may not be necessary to obtain a measure of TFVdp that is related to activity. The ability to describe a concentration-response relationship allowed identification of target concentrations of TFVdp in specific compartments so providing a means to ensure that different dosing strategies result in adequate *in vivo* TFVdp ranges for *ex vivo* HIV inhibition.

Robust dose-response correlates predicting the *in vivo* CCs in tissue, blood, rectal/vaginal fluid compartments needed for *ex vivo* suppression of HIV (i.e. EC_50,90,95_) were found using logistic regression analyses. In demonstrating these correlations, this intensive Phase 1 trial design, involving the measurement of multiple PK endpoints in multiple tissue/fluid compartments and the PD *ex vivo* assay endpoint, predicted CCs necessary for *ex vivo* suppression and, by inference, *in vivo* dosing. The *ex vivo* biopsy assay for HIV infectibility uses intact, freshly-acquired, tissue samples, providing one of the closest pre-clinical surrogates to a human efficacy clinical trial. Phase 1 rectal microbicide study designs that integrate safety, acceptability, and PK/PD measurements have the potential to provide early indications of harm and/or potential efficacy of candidate products prior to embarking on larger, longer and more expensive Phase 2B/3 trials.

When drug concentrations are independently varied, correlations between dose, *in vivo* drug concentration and infectibility can be used to predict the drug concentration needed to effectively suppress HIV infection, *ex vivo* [8]. The single doses of oral and topical drugs used here did not allow for correlations to be made with initial doses but a wide range in *in vivo* drug concentrations were found following the various treatment regimens tested. The *in vivo* drug concentrations following the 7-daily topical TFV gel treatment were quantifiable at the EC_90_ level in various compartments, and were within the range found 30 minutes post product use. The single oral TDF dose did not provide a high enough *in vivo* drug concentration for even a 50% probability of *ex vivo* suppression. The single topical TFV gel treatment provided evidence of partial suppression in some compartments but did not reach the 95% probability of suppression in any of the compartments tested.

The statistical methodology used here, and in the previous UC781 biopsy challenge trial [8], can inform on the design of future trials to reduce the number of biopsy samples required, increase power and provide relevant information from these small, early stage clinical studies. Placebo and baseline infectibility data are generally associated with high p24 variability and low statistical power [8]. Baseline and placebo data were not used (nor needed) here as the intent was to study the compartmental concentration-response relationship only following active dose(s) of the drug. Although the dose-response models found were consistent with drug-mediated virus suppression, there was only the merest indication of a lower asymptote, where suppression of HIV reached the lower limit of p24 quantification and increased drug would lead to diminished effect. Dose-ranging biopsy challenge studies are needed to provide a range of drug mediated HIV suppression in order to populate both upper and lower asymptotes of the dose-response curve. A clearly defined lower asymptote, where tissue/blood drug concentrations were correlated with p24 suppression in the explant assay, following *in vivo* exposure to a product, would provide the range of *in vivo* drug that would reliably suppress HIV in this model. Ideally, this range that would be representative of the range in *in vivo* compartmental drug concentrations found amongst users of the product.

The dose-response relationships reported here are specific to the TDF 300 mg tablet, the TFV 1% gel study product and the dosing regimens used. The EC_50,90,95_ levels calculated cannot be easily extrapolated to other doses, drugs and treatment regimens but do provide an efficacy endpoint, expressed in units of *in vivo* PK parameters, that could be comparable between studies. In the CHARM-01 study (ClinicalTrials.gov: NCT01575405) the correlations found in the RMP-02/MTN-006 study described in this paper will be reassessed for three different formulations of TFV 1% gel to evaluate whether re-formulation impacts the clear dose-response relationships found with the formulation of TFV 1% gel used in the RMP-02/MTN-006 study. For example, while compartment concentrations of TFVdp in CD4^+^_PBMC_ and CD4_Total_ were non-detectable at the time points where blood levels were paired with p24 biopsy measurements (the only time points reported here), there were detectable blood levels at sampling periods post exposure that were not paired with biopsy sampling. These are reported in our concurrently submitted manuscript comparing timing of drug detection in multiple compartments from the same Phase 1 trial [15]. Although these derivations apply only to TFV and the particular formulations and delivery methods tested, the analytical framework can be applied to many promising candidate microbicides.

The high titer of HIV infection used here (HIV_1BaL_; 10^4^ TCID_50_), far in excess of the titer found in semen [17], was previously found to reduce inter- and intra- subject variability in this tissue assay compared to a lower 10^2^ TCID_50_ titer [8]. The risk of transmission during anal intercourse has been estimated at 0.65–1.7% [17], so perhaps it is not surprising that the use of higher titer inoculants than those found *in vivo* result in higher infectibility rates *ex vivo* (∼60% of explants HIV_1BaL_10^2^ TCID_50_ [7]) in this model system. Drug treatments that reliably suppress such high HIV titers *ex vivo* set a high bar for drug mediated efficacy assays, and may overestimate the drug dose needed *in vivo* to prevent acquisition of HIV infection. The choice of isolate could increase the power of these analysis methods if a primary or mucosal-derived virus isolate was found to reliably infect mucosal tissue at lower, more virologically relevant infectious titers. Dose-ranging, use of sampling time points that reflect the known pharmacokinetic profile in each compartment, and use of more virulent HIV isolates may provide more reliable and valid dose-response analytics and derived results.

In summary, the TFVdp MMC compartment (CD4+, CD4− and Total) provided the best surrogates for biopsy infectibility and the 7-daily topical TFV gel treatment provided the strongest PK profile for HIV suppression. Although the sample size here was relatively small (N = 18, where only N = 12 received the microbicide gel formulation), the dose-response models reached statistical significance showing that meaningful, informative findings can result from this type of small, intensive PK/PD study.

## Supporting Information

File S1 **Supplemental Information.**
(DOCX)

Checklist S1 **CONSORT Checklist.**
(DOC)

Protocol S1 **Trial Protocol.**
(PDF)
