# Supplementary material for: Correlation between Compartmental Tenofovir Concentrations and an Ex Vivo Rectal Biopsy Model of Tissue Infectibility in the RMP-02/MTN-006 Phase 1 Study
Source: PLoS One. 2014 Oct 28;9(10):e111507. doi: 10.1371/journal.pone.0111507 (PMC4211741; doi:10.1371/journal.pone.0111507)
Supplement: File S1 — Supplemental Information. (DOCX) [file pone.0111507.s001.docx]

Supplemental Information

Total number of detectable, non-detectable and missing paired CC:p24 measurements .

| Compartment  (measured unit) | Post Single Oral  (V3, 5 & 6)  18 (n) x 3 (V) = 54  *4 (f) x 3 (V) = 12 | |  | Post Single Topical  (V7, 9 &10)  12 (n) x 3 (V) = 36  *2 (f) x 3 (V) = 6 | |  | Post 7-Daily Topical  (V12)  12 (n) x 1 (V)= 12  *2 (f) x 1 (V) = 2 | |  | Total  CC:p24 pairs | | Total Possible  CC:p24 pairs^§^ |
| --- | --- | --- | --- | --- | --- | --- | --- | --- | --- | --- | --- | --- |
|  | Detectable | ND  (missing) |  | Detectable | ND  (missing) |  | Detectable | ND  (missing) |  | Detectable | ND  (missing) |  |
| **TFV**  Rectal Fluid  (ng/sponge) | 38 | 15 (1) |  | 27 | 6 (3) |  | 12 | 0 |  | 77 | 21 (4) | 102 |
| Vaginal Fluid*  (ng/sponge) | 6 | 6 |  | 1 | 4 (1) |  | 2 | 0 |  | 9 | 10 (1) | 20 |
| Plasma  (ng/ml) | 45 | 8 (1) |  | 16 | 17 (3) |  | 12 | 0 |  | 73 | 25 (4) | 102 |
| Tissue  (ng/mg) | 14 | 38 (2) |  | 10 | 23 (3) |  | 10 | 2 |  | 34 | 63 (5) | 102 |
| **TFV-DP**  Tissue  (fmol/mg) | 10 | 42 (2) |  | 17 | 16 (3) |  | 12 | 0 |  | 39 | 58 (5) | 102 |
| CD4^-^_MMC_  (fmol/million) | 17 | 36 (1) |  | 17 | 16 (3) |  | 11 | 1 |  | 45 | 53 (4) | 102 |
| CD4^+^_MMC_  (fmol/million) | 10 | 43 (1) |  | 13 | 20 (3) |  | 9 | 3 |  | 32 | 66 (4) | 102 |
| Total_MMC_  (fmol/million) | 4 | 49 (1) |  | 14 | 19 (3) |  | 9 | 2 (1) |  | 27 | 70 (5) | 102 |
| CD4^-^_PBMC_  (fmol/million) | 9 | 44 (1) |  | 1 | 32 (3) |  | 0 | 12 |  | 10 | 88 (4) | 102 |

Where:

ND = non-detectable

n = subjects, f = female subjects and V = visits.

^§^ includes pairs missing either or both CC and p24 measurements
